# Guide for Using Medical Batteries

**Published:** 1867-03

**Authors:** 


Guide for Using Medical Batteries: Showing the Most Approved Apparatus, Methods, and Rules for the Medical Employment of Electricity in the Treatment of Nervous Diseases. By ALFRED C. GARRATT, M.D., Fellow of Mass. Med. Society, and Member of the Amer. Med. Association. Philadelphia: LINDSAY & BLAKISTON. 1867. For sale by W. B. KEEN & Co., 148 Lake Street, Chicago. pp. 180. Price $2.00.

This is an abridgement of the large work on Medical Applications of Electricity, by the same author. The present volume will be found very convenient for study and reference, both by students and practitioners. It gives full descriptions and illustrations of the various apparatus for applying electricity in the treatment of disease, and the best methods of using them.

